# IL-4, a direct target of miR-340/429, is involved in radiation-induced aggressive tumor behavior in human carcinoma cells

**DOI:** 10.18632/oncotarget.13561

**Published:** 2016-11-24

**Authors:** Eun Sook Kim, Young Eun Choi, Su Jin Hwang, Young-Hoon Han, Myung-Jin Park, In Hwa Bae

**Affiliations:** ^1^ Division of Basic Radiation Bioscience, Korea Institute of Radiological & Medical Sciences, Seoul, Republic of Korea

**Keywords:** radiation, interleukin-4, miR-340/429, tumorigenicity, metastasis

## Abstract

Radiotherapy induces the production of cytokines, thereby increasing aggressive tumor behavior. This radiation effect results in the failure of radiotherapy and increases the mortality rate in patients. We found that interleukin-4 (IL-4) and IL-4Rα (IL-4 receptor) are highly expressed in various human cancer cells subsequent to radiation treatment. In addition, IL-4 is highly overexpressed in metastatic carcinoma tissues compared with infiltrating carcinoma tissues. High expression of IL-4 in patients with cancer is strongly correlated with poor survival. The results of this study suggest that radiation-induced IL-4 contributes to tumor progression and metastasis. Radiation-induced IL-4 was associated with tumorigenicity and metastasis. IL-4 expression was downregulated by miR-340 and miR-429, which were decreased by ionizing radiation (IR). Radiation-regulated miR-340/429-IL4 signaling increased tumorigenesis and metastasis by inducing the production of Sox2, Vimentin, VEGF, Ang2, and MMP-2/9 via activating JAK, JNK, β-catenin, and Stat6 *in vitro* and *in vivo*. Our study presents a conceptual advance in our understanding of the modification of tumor microenvironment by radiation and suggests that combining radiotherapy with genetic therapy to inhibit IL-4 may be a promising strategy for preventing post-radiation recurrence and metastasis in patients.

## INTRODUCTION

Radiotherapy is one of the leading therapeutic options for cancer patients. Even though ionizing radiation (IR) is cytotoxic to cancer cells, recent studies have reported the paradoxical effect of IR promoting the malignant cell phenotype in various cancer types, including lung [1], hepatocellular carcinomas [2], and gliomas [3]. Moreover, many studies utilizing animal models have demonstrated that IR administered to primary tumors accelerates their spread and formation of distant metastases *in vivo* [4]. As the understanding of radiobiology has improved, investigators have sought to determine the basis for the radio-resistance of tumor cells that underlies tumor recurrence and treatment failure [5–10]. Previous studies have reported that radiotherapy can activate cytokine production [11] and cytokine regulated cellular radio-sensitivity [12]. In addition, IR-induced modification of tumor microenvironment contributes to cancer metastasis [13]. Interleukin-4 (IL-4), known as a T helper type 2 (TH2), suppresses cancer-directed immune surveillance and increases tumor metastasis [14]. Several studies have reported that IL-4 is primarily involved in the promotion of differentiation, proliferation [15], and survival of epithelial tumor cells through its interaction with IL-4Rα [16]. Increased expression of IL-4 and IL-4 receptor (IL-4Rα) has been reported in several cancer cell types, including breast, ovarian, colon, lung, and thyroid cancers [16–18]. In addition, tumor derived IL-4 can stimulate tumor-associated macrophages and promote proliferation, survival, and metastasis of tumor cells [19]. These studies suggest the potential of IL-4/IL-4Rα as a prognostic biomarker for cancer patients or therapeutic target [16]. However, the IR-induced microenvironment modification effect of IL-4 signaling on tumorigenicity, stemness maintenance, and metastasis of cancer cells has not been fully established. Here, we demonstrated that IR-induced IL-4 enhances epithelial-mesenchymal transition (EMT), migratory potential, invasiveness, angiogenesis, stemness, and metastasis of cancer cells or xenograft model. We also confirmed that IR-induced aggressive phenotypes were inhibited by IL-4 siRNA or anti-IL-4 antibody.

MicroRNAs (miRNAs) act as regulators of gene expression at the post-transcriptional level by binding to the 3′-untranslated regions (3′-UTRs) of specific mRNAs [20] and play important roles in development, proliferation, differentiation, and apoptosis [21]. It has been shown that miRNAs can act as oncogenes or tumor suppressor genes, and aberrant expression of miRNAs occurs in various tumors [22]. In this study, we screened for miRNAs that specifically target IL-4 and selected miR-340 and miR-429. We described that combining radiotherapy with IL-4-inhibiting treatment, including miR-340 and miR-429, decreased IR-induced aggressive tumor behavior. Therefore, our studies with selected miRNA-340/429, which targeted IL-4, might be a potential approach for cancer treatment.

## RESULTS

### IR induces strong expression of IL-4 and IL-4Rα in human cancer cells

**Figure 1:**
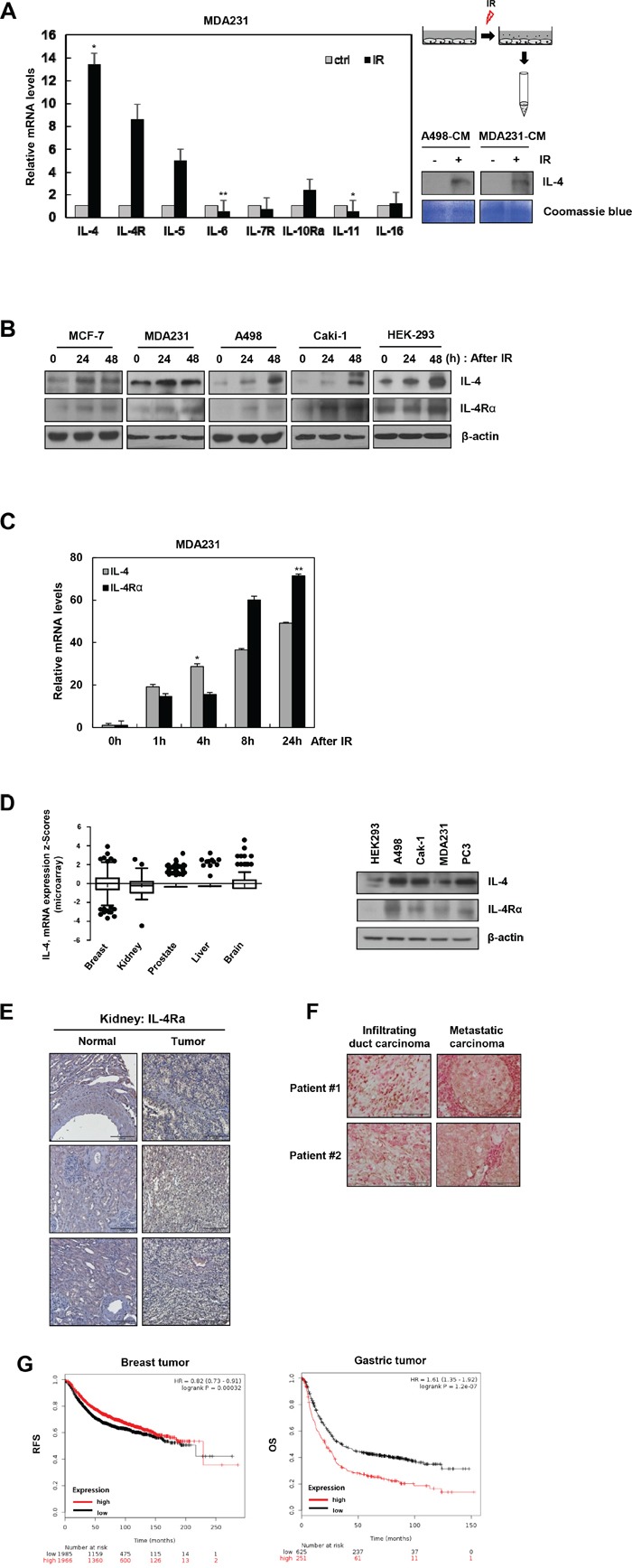
IR induces IL-4 and IL-4Rα expression in cancer cell lines **A**. Left, mRNA levels of cytokines and receptors were measured in MDA-MB-231 cells by qRT-PCR after exposure to IR (5 Gy) for 1 hour. The data are presented as the mean S.D. (* *P* < 0.05, ** *P* < 0.005, Student's t-test). Right, experimental scheme describing the collection of conditioned media (CM) from A498 or MDA-MB-231 cells treated with IR, conditioned media (CM) were collected for 3 days. The level of secreted IL-4 was checked by Western blot analysis. Coomassie staining used as a loading control. **B**. After IR treatment for 24 and 48 hours, expressions of IL-4 and IL-4Rα were measured by Western blot analysis. β-actin always served as a loading control. **C**. The mRNA levels of IL-4 and IL-4Rα in MDA-MB-231 cells were measured at 1, 4, 8, and 24 hours after treatment with IR. The data are presented as the mean S.D. (* *P* < 0.05, ** *P* < 0.005, Student's t-test). **D**. Left, IL-4 mRNA expression data in cancer patients were obtained from the TCGA microarray dataset and showed as box-and-whisker plots. Right, IL-4 protein level was also measured in various cancer cells. **E**. IL-4Rα is upregulated in all 50 renal carcinomas tissues compared with 9 normal tissues (SuperBioChips array slides, CL2) on based on IHC (Scale bar, 200 μm). **F**. IHC analysis of IL-4 expression in human breast cancer patient tissues (Superbiochips array slides, CBA4), (Scale bar, 100 μm). **G**. Kaplan-Meier analysis of IL-4 levels and survival in cancer patients from KM plotter.

IR, together with chemotherapy and surgery, is often used as a cancer therapy strategy [23–25]. However, this treatment modality alters the microenvironment of the tumor as well as distant tissues, affecting multiple cellular responses, tissue remodeling [26, 27], and cancer metastasis [27]. To detect the harmful effects of IR, we measured, using qRT-PCR, the mRNA levels of IR-induced cytokines (IL-4, IL-5, IL-6, IL-11, and IL-16) and receptors (IL-4Rα, IL-7R, and IL-10Rα), which are crucial causative agents of IR-induced microenvironmental changes in the breast cancer cells, MDA-MB-231. After IR treatment, IL-4, IL-4Rα, IL-5, IL-10Rα, and IL-16 mRNA levels increased, whereas IL-6, IL-7R, and IL-11 levels decreased. Especially, IL-4 and IL-4Rα mRNAs were highly upregulated by IR in MDA-MB-231 (Figure 1A, left) as well as in A498 cells (Supplementary Figure S1). The level of secreted IL-4 was also dramatically higher in the conditioned media from IR-treated cells compared to that from non-treated cells (Figure 1A, right). Expression of IL-4 and IL-4Rα proteins was upregulated by IR treatment in various cancer cell lines, including MCF-7, MDA-MB-231, A498, Caki-1, and HEK-293 cells, suggesting that this phenomenon is generalizable (Figure 1B). To further confirm, MDA-MB-231 cells were treated with IR for 1, 4, 8, and 24 h. As shown in Figure 1C, mRNA levels of IL-4 and IL-4Rα increased in a time-dependent manner.

### Elevated IL-4 expression in various tumor tissues has negative correlation with the survival rate of patients

The Cancer Genome Atlas (TCGA) dataset shows that the expression of IL-4 mRNA is upregulated in various cancer patients (Figure 1D, left). IL-4 and IL-4Rα were also highly expressed in several types of cancer cells (Figure 1D, right). In addition, we showed that IL-4Rα protein was upregulated in renal cancer tissues compared to that in adjacent normal tissues (Figure 1E). Interestingly, IL-4 levels were especially enhanced in metastatic carcinoma tissues compared to those in breast duct carcinoma tissues (Figure 1F). These results suggest that tumor-derived IL-4 can act as a metastatic factor that affects tumor microenvironment. IL-4 expression has been shown to be associated with increased recurrence and reduced survival in renal cancer patients [28]. To investigate the clinical significance of IL-4 expression in different cancers, overall survival data were computed from the open source KM plotter [29]. High expression of IL-4 resulted in worse survival rate of breast and gastric cancer patients (Figure 1G). These data suggest that IL-4 is a potential therapeutic target for cancer therapy.

### IL-4 is involved in IR-induced EMT, migration, invasion, angiogenesis, and stemness maintenance in human cancer cells

**Figure 2:**
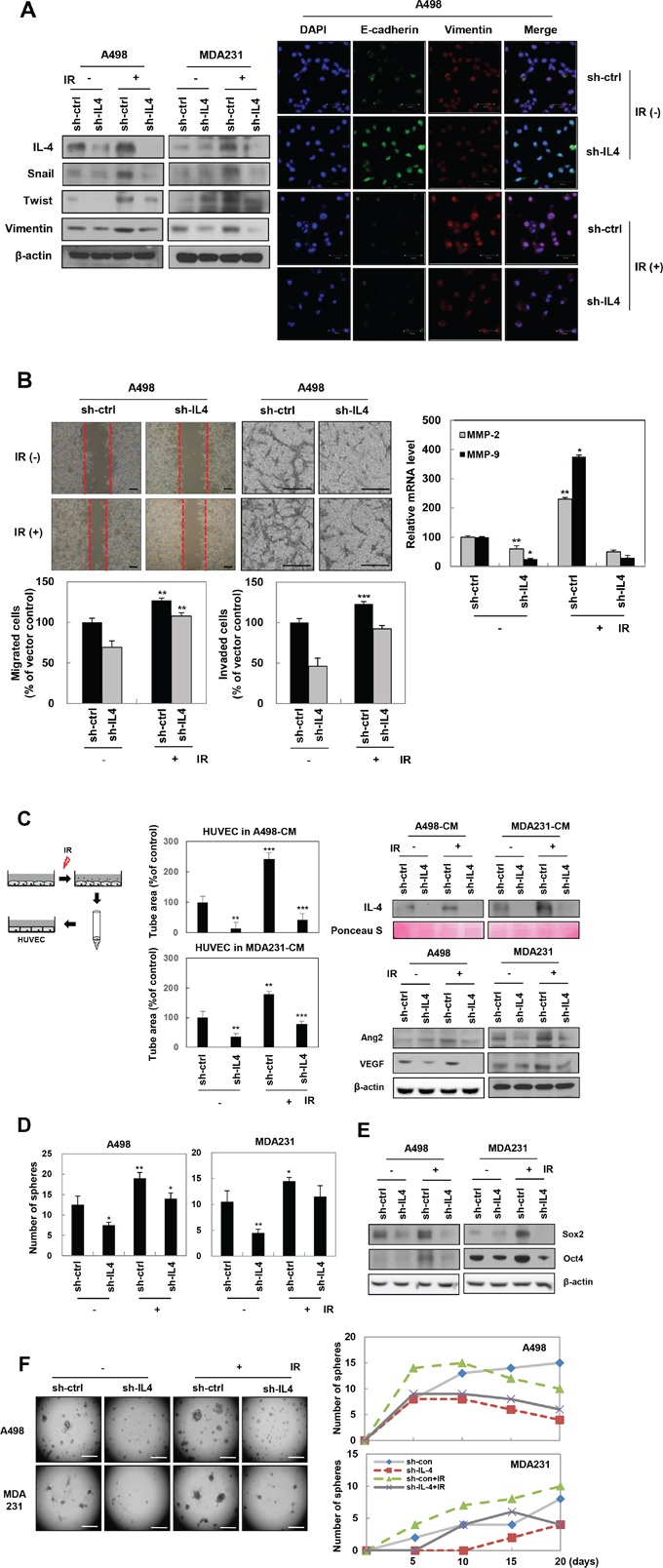
IL-4 is involved in IR-induced EMT, migration, invasion, angiogenesis, and stemness maintenance in human cancer cells A498 and MDA-MB-231 cells were transfected with shRNA IL-4 or exposed by IR. **A**. Left, after 24 hours, mesenchymal markers proteins were detected by Western blot analysis. Right, E-cadherin (Green), Vimentin (Red), and DAPI (Blue) expressions were determined by immunofluorescence staining (Scale bar, 50 μm). **B**. Cells treated as indicated were examined for invasion assays and migration assays. Left, Cells of scratched area were stained and counted after 18 hours. These experiments were performed in triplicate and the mean value was different from controls, significantly. Middle, cells were seeded onto Matrigel-coated transwell inserts. After 18 hours, the cells on the bottom side of filters were stained and counted (Scale bar, 100 μm). Right, MMP-2 and MMP-9 mRNA levels were checked in IR treatment or IL-4 knockdown A498 cells by q-RT PCR. The data are presented as the mean S.D. (** *P* < 0.005, *** *P* < 0.0005, Student's t-test). **C**. Left, experimental scheme describing the collection of conditioned media (CM) from IL4-knockdowned or IR-treated cells for 3 days. Middle, after HUVECs were treated with CM as indicated, prepared cells seeded onto Matrigel-coated 96 well plates (1 × 10^4^ cells/well), and then monitored for 6 hours to assess tube formation ability (Scale bar, 100 μm). The data are presented as the mean S.D. (** *P* < 0.005, *** *P* < 0.0005 Student's t-test). Right top, levels of secreted IL-4 in CM were verified by Western blot analysis. Ponceau S staining used as a loading control. Right bottom, angiogenesis-related proteins were detected in IL4-knockdowned or IR-treated cells by Western blot analysis. **D**. Sphere formation assays were conducted in IL4-knockdowned or IR-treated cells for 5∼10 days. These experiments were performed in triplicate and the mean value was different from controls, significantly (Scale bar, 100 μm). The data are presented as the mean S.D. (* *P* < 0.05, ** *P* < 0.005, Student's t-test). **E**. Expressions of cancer stem-like cells marker proteins were determined by Western blot analysis. **F**. IL4-knockdowned or IR-treated cells seed on pre-coat 24-well plate with 60 μL of Matrigel and grow cells for 20 days. Photo (left) and graph (right) were taken every 5 days. These experiments were performed in triplicate (Scale bar, 500 μm).

To determine whether IL-4 is involved in the acquisition of IR-induced tumorigenic properties in cancer cells, we evaluated the effect of IL-4 on IR-induced tumor progression, including enhancement of mesenchymal-related protein expression, migratory potential, invasiveness, angiogenesis, and stemness maintenance. Knockdown of IL-4, using small hairpin RNA (shRNA) against IL-4 in A498 and MDA-MB-231 cells, decreased IR-induced expression of the mesenchymal marker proteins such as Snail, Twist, and Vimentin, as demonstrated by Western blot analysis (Figure 2A, left) and immunofluorescence staining (Figure 2A, right). These results were supported by changes in the cell morphology exhibited by some of the cancer cells at the invasive edge, such as the epithelial cancer cells losing the cuboidal shape and changing to an elongated and fibroblastic shape. sh-IL-4 transfected A498 cells exhibited a decrease in the IR-induced migratory (Figure 2B, left) and invasive potentials (Figure 2B, middle) by attenuating the mRNA levels of the matrix metalloproteinases (MMPs), MMP-2 and MMP-9 (Figure 2B, right).

To evaluate the effect of IR-induced IL-4 on angiogenesis, we performed tube formation assay *in vitro*, which is widely used as a model for the reorganization stage of angiogenesis [30]. For the assay, we collected conditioned media (CM) from A498 and MDA-MB-231 cells transfected with sh-IL4 or treated with IR (Figure 2C, left). Each CM that contained the factors secreted by the cells was analyzed for levels of secreted IL-4 and used for tube formation assays. The level of IL-4 was dramatically higher in CM from IR-treated cells compared with that in CM from non-treated or sh-IL-4-transfected cells. The level of IL-4 in CM from IR-treated cells after sh-IL4-transfection was effectively attenuated, compared with that in CM from only IR-treated cells (Figure 2C, right top). Tube formation in HUVECs suspended in each of the two CMs showed positive correlation with the level of secreted IL-4 (Figure 2C, middle). Expression of the angiogenesis-related proteins, angiopoietin-2 (Ang-2) and vascular endothelial growth factor (VEGF), was downregulated in IL4-knockdown cells exposed to IR treatment, compared with only IR treated cells (Figure 2C, right bottom). All these results taken together indicate that IL-4 is involved in IR-induced acquisition of tumorigenic properties by human cancer cells.

To investigate the role of IR-induced IL-4 in stemness maintenance, A498 and MDA-MB-231 cells were transfected with sh-IL4 or treated with IR, and the growth was measured using a sphere-forming assay and three-dimensional culture assays. These assays showed that IR-induced ability of sphere and colony formation was decreased in sh-IL-4 knockdown cell lines (Figure 2D and 2F). Moreover, the attenuated sphere forming ability of IL4-knockdown cells was accompanied by the downregulation of Sox2 and Oct4 (Figure 2E), which are known as markers of breast and renal cancer stem cells [31–34]. These results suggest that IL-4 mediates IR-induced maintenance of stemness in a stem-like cell population of human cancer cells.

### IL-4 promotes EMT, migration, invasion, angiogenesis, and stemness by activating the JAK/JNK/β-catenin/Stat6 signaling pathway in human cancer cells

**Figure 3:**
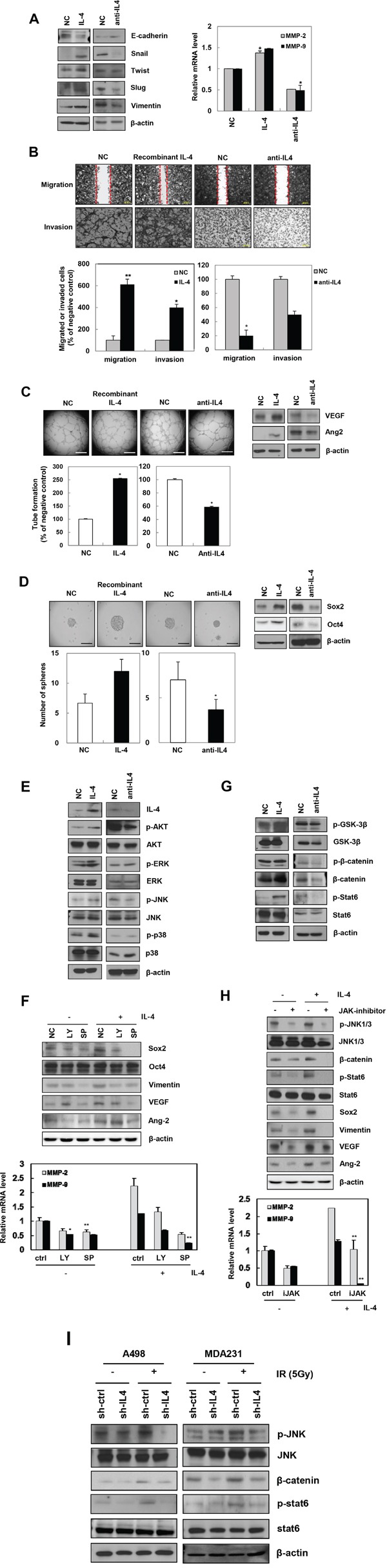
Recombinant IL-4 or neutralization of IL-4 using anti-IL-4 regulates EMT, migration, invasion, angiogenesis, and stemness in cancer cells **A**. After treatment of recombinant IL-4 (0.5 ng/mL) or anti-IL-4 antibody (10 μg/mL) for 24 hours in A498 cells, mesenchymal markers proteins and MMP-2/9 mRNA levels were determined by Western blot analysis (left) and q-RT PCR (right), respectively. The data are presented as the mean S.D. (* *P* < 0.05, Student's t-test). **B**. Top, after treatment of recombinant or antibody of IL-4, migration assay was performed after 18 hours. Bottom, cells were seeded onto Matrigel-coated transwell membranes to detect the invaded cells for 18 hours, stained and counted (Scale bar, 100 μm). The data are presented as the mean S.D. (* *P* < 0.05, ** *P* < 0.005, Student's t-test). **C**. Ability of tube formation and expression of angiogenesis-related factors were checked in A498 using IL-4 recombinant or anti-IL-4 antibody, respectively (Scale bar, 500 μm). The data are presented as the mean S.D. (* *P* < 0.05, Student's t-test). **D**. Stemness - ability of sphere forming and expression of cancer stem-like cells marker proteins was determined by sphere formation assay (Scale bar, 100 μm) and Western blot analysis. The data are presented as the mean S.D. (* *P* < 0.05, Student's t-test). **E**. A498 cells were treated with recombinant IL-4 or anti-IL-4 antibody and the expression of the indicated signaling molecules was measured by Western blot analysis. **F**. After treatment of inhibitors of PI3K (LY; LY294002, 10 μM), and JNK (SP; SP60025, 10 μM), IL-4 signaling-related molecules were determined by Western blot analysis (top) and MMP-2/9 mRNA levels were quantified by qRT-PCR in A498 cells (bottom). **G**. Upstream signaling factors induced by recombinant IL-4 or anti-IL-4 antibody were checked by Western blot analysis. **H**. After treatment of JAK (1 μM) inhibitor, IL4-induced upstream signaling molecules were determined by Western blot analysis (top) and MMP-2/9 mRNA levels were quantified by qRT-PCR (bottom) in A498 cells. **I**. IL-4 signaling factors regulated by IR were detected by Western blot analysis.

To confirm the effect of IL-4 on cancer cell phenotype, we used a recombinant IL-4 (0.5 ng/mL) or an anti-IL-4 antibody (10 μg/mL) and examined changes in mesenchymal trait, migration, invasion, angiogenesis, and stemness in A498 cells. Exogenous IL-4 induced EMT-related proteins. Specifically, Snail, Twist, Slug, and Vimentin levels were increased, whereas E-cadherin was reduced. Conversely, neutralizing IL-4 activity with anti-IL-4 antibody decreased the expression of mesenchymal markers (Figure 3A, left). Exposure of A498 cells to recombinant IL-4 increased their migratory and invasive potential through transcriptional regulation of genes encoding MMP-2 and MMP-9, whereas anti-IL-4 antibody decreased the migration and invasion (Figure 3A, right and 3B). There was no proliferation under these experimental conditions (data not shown). Tube-forming ability and expression of angiogenesis-related factors, VEGF and Ang2, were enhanced by recombinant IL-4, but were inhibited by anti-IL-4 antibody (Figure 3C). In addition, sphere formation ability and expression of the cancer stem-like cell proteins, Sox2, and Oct4 were upregulated by recombinant IL-4 and downregulated by anti-IL-4 antibody in A498 cells (Figure 3D), similar to the phenomena exhibited by MDA-MB-231 cells (Supplementary Figure S2A and 2B). These data suggest that IL-4 enhances migratory potential, invasiveness, angiogenesis, and stemness maintenance in human cancer cells.

We next used recombinant IL-4 and anti-IL-4 antibody to evaluate the signaling mechanism by which IL-4 mediates IR-induced tumor progression in human carcinomas. Recombinant IL-4-treated cells dramatically activated Akt and JNK (c-Jun N-terminal kinase). However, there was no change in p38 and ERK (Extracellular signaling-regulated kinase) by IL4 treatment (Figure 3E). Using inhibitors of Akt and JNK, we confirmed that exogenous IL-4 increased Sox2, Vimentin, VEGF, Ang2, and MMP-2/9 by activating Akt and JNK. Especially, IL-4-induced factors mentioned above were dramatically downregulated by JNK inhibitor (Figure 3F). Overexpressing or inhibiting IL-4 to identify IL-4 signaling-related transcription factors, dramatically altered Stat6 (signal transducer and activator of transcription 6) and β-catenin expression (Figure 3G). Therefore, we used JAK (Janus kinases) inhibitor to examine Stat6 signaling pathway. We found that JAK upregulated Sox2, Vimentin, VEGF, Ang2, and MMP-2/9 by activating JNK (Figure 3H) in A498, similar to the IL-4-induced signaling exhibited by MDA-MB-231 cells (Supplementary Figure S2C). In addition, IL-4 signaling increased tumorigenic factors such as Vimentin, VEGF, and Ang2 by activation of β-catenin and Stat6 (Supplementary Figure S3). We also confirmed the role of IL-4 signaling pathway in IR-induced tumor progression using sh-IL4-transfected A498 and MDA-MB-231 cells (Figure 3I). Cumulatively, these results suggest that IR-induced IL-4 regulates tumor progression by promoting β-catenin/Stat6 via activation of JAK and JNK.

### IR-induced IL-4 promotes pulmonary metastasis of breast cancer cells in nude mice

**Figure 4:**
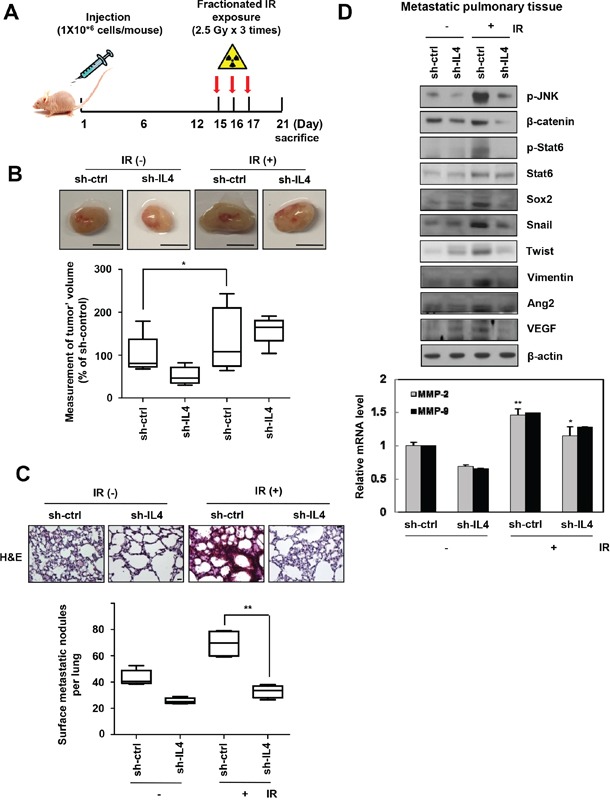
IR-induced IL-4 promotes primary breast cancer metastasis in nude mice **A**. The plan of animal experiments was described, whichsh-IL4-tranfectedmetastatic MDA-MB-231 cells were orthotopically injected into mammary fat pads of nude mice. After implantation, mice were exposed to IR 3 times per 2.5 Gy and then sacrificed at 21 days. **B**. Tumor growth (Scale bar, 5 mm) and **C**. lung metastatic nodules (Scale bar, 20 μm) were showed as box-and-whisker plots (bottom) and lung tissues of mice were stained with H&E (top) (n=5 animals for group), respectively. The data are presented as the mean S.D. (* *P* < 0.05, ** *P* < 0.005, Student's t-test). **D**. Using metastatic lung tissues of mice, IR-induced IL-4-regulated signaling molecules were detected by Western blot analysis (top) or q-RT PCR (bottom).

To determine the effect of IR-induced upregulation of IL-4 on metastasis *in vivo*, we implanted orthotopically, IL-4-knockdown or negative control of metastatic MDA-MB-231 cells into nude mice and then exposed them to IR (2.5 Gy) on days 15, 16, and 17. Mice were sacrificed 21 days after implantation (Figure 4A). The fractionated radiation system is advantageous because of the fewer toxic effects on normal cells. Then we selected the fractionated dose to avoid exposing the mice to the acute dose [35]. Using wound healing assay, invasion assay, and sphere formation assay, we showed that the biological events occurring in the cells exposed to a single dose (5 Gy) were similar to those in the cells receiving fractionated dose (2.5 Gy × 3 times) (Supplementary Figure S4). The size of primary breast tumors and number of nodules in the lung were lower in mice injected with IL4-knockdown cells compared with those in mice injected with wild type cells (control mice), which showed a dramatic increase in both the size of primary breast tumors and number of metastatic lung nodules following fractionated IR treatment (Figure 4B and 4C). A histological examination of metastatic pulmonary tissues of the mice, using hematoxylin and eosin (H & E) staining, showed low density in mice injected with sh-IL4-transfected cells compared to those from control mice (Figure 4C). The metastatic pulmonary tissue of fractionated IR treated mice also showed intense H & E staining compared to that of control mice and IR-treated mice injected with IL-4-knockdown cells. In addition, mesenchymal markers, invasiveness related enzymes, angiogenesis-related proteins, and the cancer stem-like cell marker proteins were upregulated by activating the JNK/β-catenin/Stat6 signaling axis in metastatic pulmonary tissues (Figure 4D). Immunohistochemistry showed that IR-induced IL-4 increased the expression of major components related to p-Stat6, mesenchymal trait marker, Vimentin; invasiveness marker, MMP-9; stemness maintenance marker, Sox2; and tumor proliferation marker, Ki-67 (Supplementary Figure S5). In addition, expression of β-catenin, Oct4, MMP-2, Ang2, and VEGF was also increased slightly (data not shown). Thus, IR-induced IL-4 enhances primary breast cancer metastasis to lung by stimulating the expression of components related to mesenchymal traits, invasiveness, angiogenesis, stemness maintenance, and proliferation. These results provide compelling evidence that IR-induced IL-4 expression mediates metastatic capacity of cancer cells *in vivo*.

### IL-4 is a direct target of miR-340 and miR-429 which downregulated by IR

**Figure 5:**
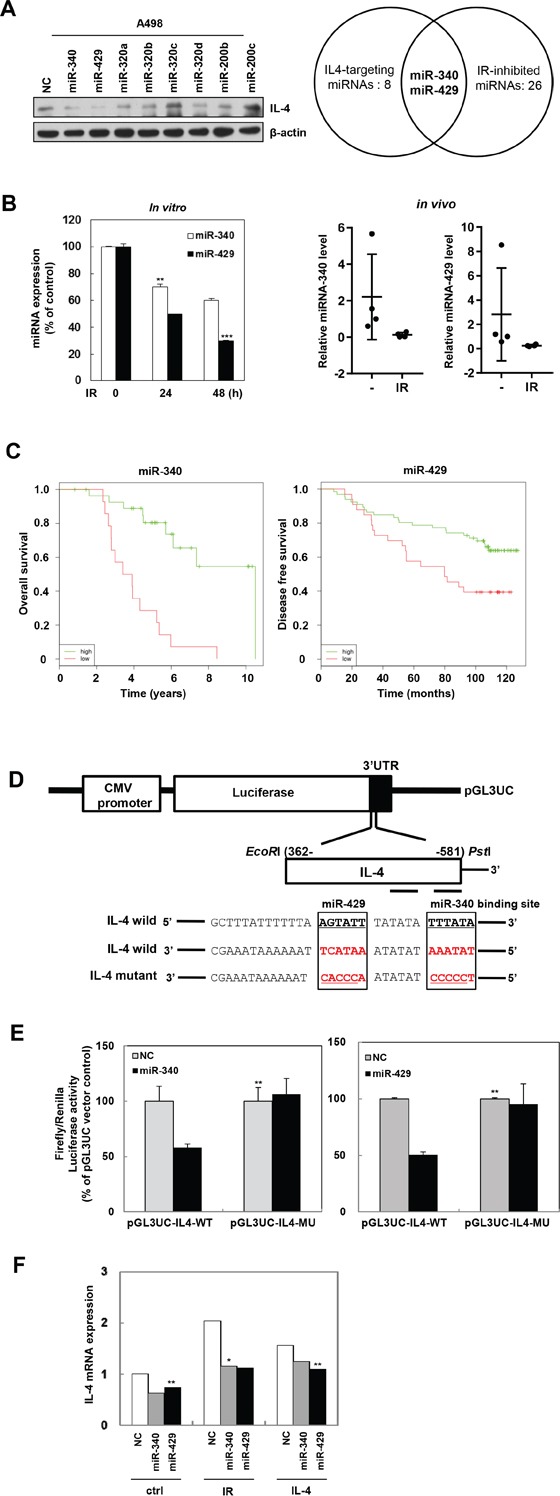
IL-4 is a direct target of miR-340/429 **A**. IL-4 targeting-eight miRNAs were selected using miRNA prediction sites and were confirmed by Western blot analysis (left). IR-inhibited 26 miRNAs were picked according to the previous study of Weidhaas and Chaudhry group [36, 37] (right). miR-340 and miR-429 were selected as IR-inhibited and IL-4-targeting miRNAs for further investigation. **B**. After a single or fractionated IR exposure to A498 cells (5 Gy) (left) or mouse (2.5Gy x 3 times) (right) respectively, the expression of miR-340 or miR-429 in cells or mouse pulmonary tissues was quantified by qRT-PCR. The data are presented as the mean S.D. (** *P* < 0.005, *** *P* < 0.0005, Student's t-test). **C**. Kaplan-Meier analysis ofbreast cancerpatients was available in the MIRUMIR. Left, overall survival of miRNA-340 (probe ID: 29872). Right, disease-free survival of miRNA-429 (probe ID: has-mir-429). **D**. Structure of reporter constructs containing IL-4 3’UTR downstream of the luciferase ORF. pGL3UC-IL4 vectors containing the wild-type miR-340/429 binding site or a non-binding mutant were constructed. **E**. Luciferase activities for reporters with wild or mutant 3’UTR of IL-4 which co-transfected with the indicated oligonucleotides were measured in the A498 cells. The data are presented as the mean S.D. (** *P* < 0.005, Student's t-test). **F**. IL-4 mRNA levels of cells treated as indicated were detected by qRT-PCR. The data are presented as the mean S.D. (* *P* < 0.05, ** *P* < 0.005, Student's t-test).

To identify miRNAs that control the expression of IL-4 and are downregulated by IR, we used miRNA target prediction tools such as Targetscan and miRanda. A total of eight miRNAs (Figure 5A, left) were selected for further investigation and expression levels of IL-4 protein were also verified by western blot analysis. From the 26 miRNAs that are downregulated by IR per earlier reports [36, 37], we focused on miR-340 and miR-429 (Figure 5A, right). We confirmed that IR suppressed the miR-340 and miR-429 mRNA expression in A498 cells in a time dependent manner (Figure 5B, left) as well as mouse pulmonary tissues which exposed by fractionated IR (Figure 5B, right). We also showed that miR-340/429 expression is positively correlated with the survival rate of patients (Figure 5C), using Kaplan-Meier analysis [38]. To verify if IL-4 was a direct target for miR-340 and miR-429, we constructed the 3′-UTR reporter plasmids containing full length IL-4 3′-UTR with wild type (wt) or mutated (mut) miR-340/429 binding site (Figure 5D). Using luciferase assay, we showed that miR-340 and miR-429 could repress the expression of reporter gene containing wt 3′-UTR but not that containing mutated 3′-UTR (Figure 5E). To confirm the relationship between IR-induced IL-4 and miR-340/429, we measured IL-4 mRNA levels after treatment with IR or recombinant IL-4 in the presence or absence of synthetic miR-340 or miR-429. Both the synthetic miR-340 and miR-429 efficiently suppressed the induction of IL-4 mRNA expression by IR and recombinant IL-4 (Figure 5F). These results suggest that IL-4 is a direct target for miR-340 and miR-429.

### miR-340 and miR-429 inhibit IR- and IL-4 induced tumorigenicity

**Figure 6:**
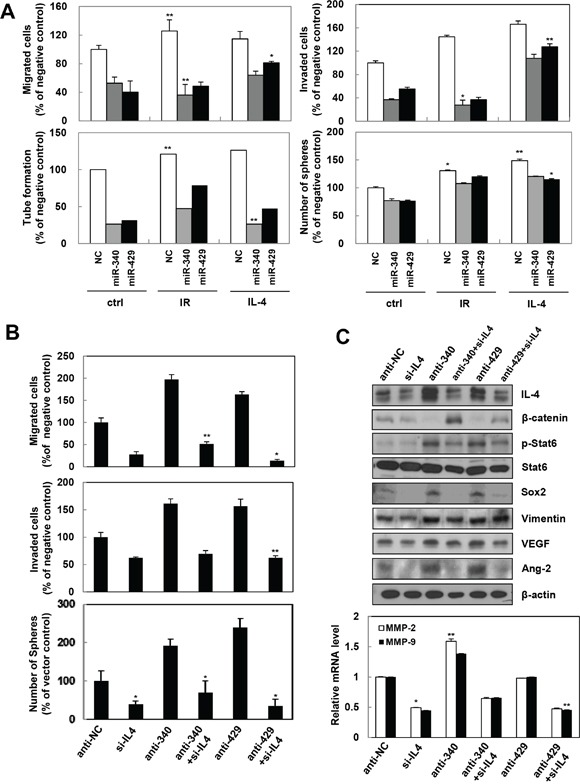
miR-340/429 mimics inhibit IR or recombinant IL-4-induced aggressive properties in the cancer cells **A**. After cells transfected with miR-340/429 mimics and treated with IR or recombinant IL-4, A498 and HUVEC cells subjected to migration, invasion, sphere-formation, and tube formation assays, respectively. The data are presented as the mean S.D. (* *P* < 0.05, ** *P* < 0.005, Student's t-test). A498 cells were transfected with each indicated oligonucleotide for migration, invasion, and sphere-formation assays **(B)**, Western blot analysis (**C**, top), and qRT-PCR (C, bottom). The data are presented as the mean S.D. (* *P* < 0.05, ** *P* < 0.005, Student's t-test).

Gain and loss-of-function approaches were used to determine whether miR-340/429 were involved in IR or IL-4 induced tumorigenicity. Both the synthetic miR-340 and miR-429 mimics inhibited IR or recombinant IL-4 induced migration, invasion, tube formation, and sphere formation abilities in A498 or HUVECs (Figure 6A). To confirm the function of miR-340/429 and their target, IL-4 was knocked down using IL-4 siRNA. Loss of IL-4 induced by miR-340/429 inhibitors abolished the migratory, invasive, and sphere forming abilities of the cells (Figure 6B). Knocking down IL-4 also inhibited the production of IR-induced signaling molecules associated with IL-4 such as β-catenin, Stat6, Sox2, Vimentin, VEGF, Ang2, and MMP-2/9 in A498 (Figure 6C). These data further demonstrated that miR-320/429 could directly target IL-4 to suppress tumor progression and metastasis.

**Figure 7:**
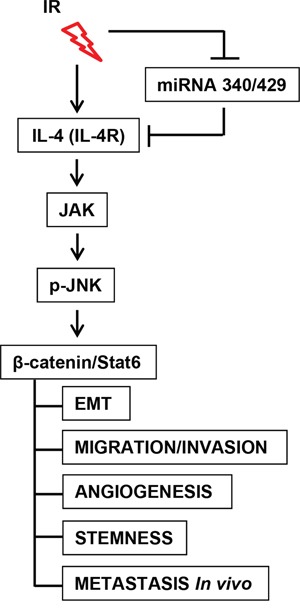
Scheme of IR-related miR-340/429-IL4-β-catenin/Stat6 signaling axis IR upregulated IL-4 expression by suppressing the expression of miR-340/429. Ultimately, IR-regulated miR-340/429-IL4-Stat6 axis was involved in tumor progression and metastasis of human cancer cells through activations of JAK and JNK.

Collectively, our results demonstrated that IR upregulates IL-4 expression by suppressing the expression of miR-340 and miR-429. Finally, IR-induced IL-4 promotes tumor progression and metastasis by increasing β-catenin/Stat6 through activation of JAK/JNK in human cancer cells (Figure 7).

## DISCUSSION

IR is often used as a therapeutic option for cancer patients in conjunction with chemotherapy and surgery [23–25]. IR evokes changes in the tumor microenvironment, altering the composition of cytokines and chemokines associated with multiple cellular responses, tissue remodeling [26, 39], and cancer metastasis [27]. To study the tumor microenvironment-related effects of IR on tumor progression and metastasis, we screened for IR-induced cytokines and found that IL-4 mRNA levels were increased dramatically after IR exposure in various cancer cell types (Figure 1A–1C). According to previous reports, IL-4 and IL-4Rα expression are increased in several types of tumor cells, suggesting the involvement of IL-4 in tumor progression and metastasis after IR exposure [16, 17, 36, 40–42]. We found that IL-4 level is significantly upregulated in tissues of cancer patients and cancer cell lines (Figure 1B and 1D). We also confirmed that IL-4Rα expression was increased in renal cancer tissues compared with that in adjacent normal cancer tissues (Figure 1E). IL-4 expression is markedly elevated in breast metastatic carcinoma (Figure 1F) and is correlated with patient survival (Figure 1G). High level of IL-4 expression is associated with increased recurrence and reduced survival in renal carcinoma patients [28]. These results suggest the potential role of IL-4 in the survival following radiotherapy. It was previously reported that IL-4 plays a critical role in the regulation of immune responses and is detected at high levels in the tumor microenvironment of cancer patients, where it is correlated with the grade of malignancy [43, 44]. Survival, proliferation, and metastasis of cancer cells are influenced by multiple factors, including cytokines in the tumor microenvironment that interact with surrounding cells and regulate complex signaling pathways [44]. In addition, IL-4 has been shown to increase the proliferation of colon, breast, head and neck, ovary, and prostate cancer cells *in vitro* [42]. However, there is no information as to how IR-induced IL-4 expression translates to tumor progression and metastasis.

Here, we report that IR-induced IL-4 signaling is strongly associated with EMT, migratory potential, invasiveness, angiogenesis, stemness maintenance, and metastasis of cancer cells (Figure 2–4). EMT facilitates important cellular actions associated with tumorigenicity and metastasis of various tumors and thus is a crucial step in cancer invasion and metastasis [43, 44]. IR-induced mitogenic signaling pathways can induce angiogenesis and an invasive phenotype in cancer cells [4], which in turn confer aggressiveness [13]. It has also been reported earlier that transition to a mesenchymal gene-expression pattern is associated with the acquisition of cancer stem cell properties [16, 17]. Conditioned media from sh-IL4 transfected cells exposed to IR had lower levels of secreted IL-4 and promoted angiogenesis in endothelial cells (Figure 2C). These results provide the evidence for IR-induced modification of tumor microenvironment.

By modulating IL-4 expression using siRNA, recombinant IL-4, or anti-IL-4 antibody, we found that IR-induced IL-4 signaling activates Akt/JNK via JAK, which in turn enhances β-catenin/Sox2/Oct4/Stat6 signaling and subsequent MMP-2/9 activation, ultimately leading to tumor progression and stemness maintenance of human cancer cells (Figure 3E–3H and Supplementary Figure S2). IL-4, as an autocrine factor, has a critical role for survival signals of cancer stem-like cells [45]. IR is known to activate Akt [4, 46] and Stat3 signaling pathways resulting in radio-resistance in cancer [5]. IL-4 also promotes migration of cancer cells via the Akt/Stat6 pathway signaling [14, 47, 48]. We found that IR-induced IL-4 signaling also promoted the growth of primary breast tumors and their metastasis to lung in nude mice by activating IL-4/JNK/β-catenin/Stat6 signaling (Figure 4A–4D and Supplementary Figure S5). Various studies utilizing animal models have demonstrated that IR administered to primary tumors accelerates their spread and formation of distant metastases [3].

Extensive research has established the role of miRNAs as important regulators of gene expression in cancer biology [49, 50]. Different miRNAs are variably expressed in different cancer types and are critically involved in regulating cell survival, apoptosis [51], proliferation, cell death, and tumorigenesis. In this study, we have identified miR-340 and miR-429 as specific miRNAs for the IL-4 directed gene regulation in cancer cells (Figure 5A–5C). We also showed that miR-340 and miR-429 directly bind the 3′-UTR of IL-4 gene and inhibit the expression IL-4 mRNA and protein (Figure 5D–5F). miR-340 and miR-429 attenuated tumorigenicity, ultimately acting as tumor suppressors (Figure 6). Thus, the gene encoding IL-4 is a direct target of miR-340 and miR-429, which normally repress the expression of IL-4. Accordingly, IR-induced downregulation of miR-340 and/or miR-429 de-represses IL-4 expression and promotes tumor progression, ultimately making IL-4 to act as a malignancy inducer. Target site prediction studies indicate that miR-429 targets and inhibits Sox2 expression as well as IL-4 expression [52].

In summary, IR affects the miR-340/429/IL-4/β-catenin/Stat6 signaling axis through activation of JAK and JNK, effectively enhancing tumor progression and metastasis of human carcinomas (Figure 7). Our findings represent a conceptual advance in the understanding of IR-induced changes in the tumor microenvironment, and suggest that a gene therapy that inhibits IL-4 combined with radiotherapy is a promising strategy for preventing aggressive tumor behavior.

## MATERIALS AND METHODS

### Cells culture

MCF-7 (human breast carcinoma), A498 (renal cell carcinoma) and Caki-1 (kidney carcinoma), PC3 (prostate carcinoma), and HEK-293 (normal kidney) cells were purchased from America Type Culture Collection (Manassas, VA, USA). Human breast cancer cells, highly metastatic MDA-MB-231 cells were kindly provided by S.J. Lee (Department of Life Sciences, Hanyang University, Korea) [35]. These cell lines were cultured in RPMI1640 or DMEM (Mediatech Inc., Manassas, VA, USA) medium containing 10% fetal bovine serum and ampicillin and streptomycin at 37°C in a humidified atmosphere of 95% air and 5% CO_2_. HUVECs (human umbilical vein endothelial cells) cultured in endothelial cell growth medium MV2 with supplementmix (Promo Cell GmbH, Heidelberg, Germany).

### Chemical reagents and antibodies

Polyclonal antibodies against p-Akt (Ser473), Akt, and p-GSK3β, and monoclonal antibodies to β-catenin and Vimentin, were purchased from Cell Signaling Technology (Danvers, MA, USA). Polyclonal antibodies to JNK, Slug, Sox2, p-Stat6, Stat6, Ang-2, and VEGF, and monoclonal antibodies to p-JNK, β-actin, and Oct4 were obtained from Santa Cruz Biotechnology (Dallas, TX, USA). Polyclonal antibody to Snail was purchased from Novus Biologicals (Littleton, CO, USA). The monoclonal antibody E-cadherin was obtained from BD Transduction Laboratories (San Jose, CA, USA). The polyclonal antibody Twist was purchased from Abcam Inc. (Cambridge, MA, USA). Monoclonal antibodies to IL-4 and IL-4Rα were obtained from R&D systems (Minneapolis, MN, USA). Anti-mouse and anti-rabbit Alexa Fluor 488 and 555-conjugated secondary antibodies were purchased from Thermo Fisher scientific (Waltham, MA, USA). Inhibitors of JAK, PI3K (LY294002), and JNK (SP600125) were purchased from Merck Millipore (Darmstadt, Germany). Recombinant IL-4 was obtained from R&D systems (Minneapolis, MN, USA).

### Plasmid DNAs

pGL3UC vector construct was kindly provided by V. N. Kim (School of Biological Sciences, Seoul National University, Korea) [53]. To perform the reporter construct, a DNA fragment of human IL-4 3′-UTR containing the putative miR-340 binding site (TTTATA, PCR product size;162 bp) and miR-429 binding site (AGTATT, PCR product size;151 bp) were amplified by PCR, and cloned into pGL3UC. The nucleotide sequences of primers for the amplification of the IL-4 3’UTR were pGL3UC-IL4-miR-429 wild type, forward, 5’-CAAGCAGCTGATCCGAT TCC-3’; reverse (wild) (underline: binding site), 5’-TGCACTGCAG**AATACT**TAAAA-3’; reverse (mutant), 5’-TGCACTGCAG**ACCCAC**T AAAA-3’; pGL3UC-IL4-miR-340 wild type, forward, 5’-CAAGCAGCTGATCCGA TTCC-3’; reverse (wild) (underline: binding site), 5’-TGCACTGCAG**TATAAA**TATAT-3’; reverse (mutant) 5’-TGCACTGCAG**TCCCCC**TATAT-3’.

### RNA oligoribonucleotides and transfection

Synthetic miRNA mimics or inhibitors were synthesized by IDT Incorporation (Integrated DNA Technologies, Inc., Coralville, Iowa, USA) as RNA duplexes designed from the sequences of miR-340 (5′-UUAUAAAGCAAUGAGACUGAUU-3′) and miR-429 (5′-UAAUACUGUCUGGUAAAACCGU-3′) using 5’-UGAAUUAGAUGG CGAUGUUTT-3’ for the negative control. The inhibitor of miR-340 or miR-429 was a 2’-*O*-methyl-modified oligoribonucleotide single strand with the sequence as miR-340, 5′-AAUCAGUCUCAUUGCUUUAUA-3′ or as miR-429 5′-ACGGUUUUACCA GACAGUAUU-3′. siRNA duplexes, shRNA, and miRNAs (20 nM) were introduced into cells for 48 hours using Lipofectamine 2000 reagent (Thermo Fisher Scientific, Grand Island, NY, USA) according to the recommended procedures. All siRNAs (β-catenin, Sox2, Oct4, and Stat6) and shRNA (sh-IL-4) were purchased from Santa Cruz Biotechnology (Dallas, TX, USA).

### Ionizing irradiation treatment of cells

Ionizing radiation (IR) was performed to 50% confluent cells in 100 mm dishes as 5 Gy and do further experiment at 24 hours after IR. Cells were then exposed to γ-rays with 137Cs γ-rays source (Atomic Energy of Canada, Ltd, Canada) with a dose rate of 3.81 Gy/min.

### Quantitative real-time PCR

Total cellular RNAs were extracted using TRIzol reagent (Molecular Research Center Inc., OH, USA) according to the manufacturer's instruction. First-strand cDNAs were synthesized using a mixture with Tetro cDNA Synthesis Kit (Bioline, London, UK) and were amplified using RCR cycler (DNA Engine Opticon^®^2 system, Bio-Rad laboratories, Inc., Herculues, CA, USA). The level of miR-340 or -429 were quantified by real-time qRT-PCR using Mir-X miRNA qRT-PCR SYBR kit (Clontech Laboratories Inc., Mountain view, CA, USA). Primer of IL-4, IL-4Rα, MMP-2/9, and miR-340/429 were purchased from as follows; IL-4 forward, 5’-GCCACCATGAGAAGGACACT-3’ and IL-4 reverse, 5’-ACTCTGGTTGGCTTCCT TCA-3’; IL-4Rα forward, 5’-CCAGTGGAGATCAGCAAGAC-3’ and IL-4Rα reverse, 5’-GGAACAGGCTCTCTGTTAGC-3’; MMP-2 forward, 5’-CATCAAGGGCATTCAGG AGC-3’; MMP-2 reverse, 5’-AGAACACAGCCTTCTCCTCC-3’; MMP-9 forward, 5’-TC GTGGTTCCA ACTCGGTTT-3’; MMP-9 reverse, 5’-GGTTTCCCATCAGCATTGCC-3’ GAPDH forward, 5’-TGCCTCCTGCACCACCAACTGC-3’, GAPDH reverse, 5’-AATGCCAGCCCCAGCGTCAAAG3’; miR-340 (5’-TTATAAAGCAATGAGACTGATT-3’) or miR-429 (5’-TAATACTGTCTGGTAAAACCGT-3’). The housekeeping gene encoding GAPDH was used as an internal control. The cycle threshold (Ct) values are similar to within 0.5 among triplicates. The primer of U6 used for normalization. The 2^-ΔΔCt^ values of miR-340/429 and U6 were calculated and the experiments were performed in triplicate.

### Western blot analysis

Cell lysates were prepared by extracting with RIPA buffer supplemented with protease inhibitors (Roche, Indianapolis, IN, USA). Total protein extract prepared from A498 or MDA-MB-231 cells was separated by SDS-PAGE and transferred to a polyvinylidene difluoride (PVDF) membrane. We performed the next steps according to the manufacturer's instruction [54].

### Wound healing assay

A498 or MDA-MB-231 cells were grown to confluence and scratched using a yellow tip. Cells were washed with pre-warmed phosphate-buffered saline (PBS) to remove cellular debris and allowed to migrate for 16-24 hours. The cells were fixed and stained with 0.05% crystal violet in 10% ethanol. The cell number of five fields in the wounded area (200 × 500 μm^2^) were counted under a light microscope (Motic Incorporation Ltd., Causeway, Hong Kong) [55].

### Transwell invasion assay

Invasion assay was performed using matrigel (BD Biosciences, San Jose, CA, USA) coated transwell chambers (8 μm pore) (Corning, Corning, NY, USA). Cells (2.5 × 10^4^ cells/well) were placed in the upper transwell chamber and medium containing 10% FBS was added to the lower chamber. The next steps were done according to the manufacturer's instructions [54]. The stained cells were counted under a light microscope.

### Reporter assay

Cell reached to around 50% confluency in 24-well culture plates and then co-transfected for 48 hours with reporter plasmid (200 ng), pRL-CMV-*Renilla* (Promega, Madison, WI, USA) plasmid (1 ng) and miRNA using Lipofectamine 2000 (Thermo Fisher Scientific, Grand Island, NY, USA). Luciferase activity was measured using dual-luciferase reporter Assay system (Promega, Madison, WI, USA) according to the manufacturer's instructions and normalized to *Renilla* luciferase activity [53]. All experiments were performed in triplicate.

### Sphere culture and sphere formation assay

A498 or MDA-MB-231 cells containing control shRNA or IL-4 shRNA was suspended in Dulbecco's modified Eagle's medium-F12 (Mediatech Inc., Manassas, VA, USA) containing B27 (1:50) (Thermo Fisher Scientific, Grand Island, NY, USA) as cancer stem cell permissive medium. For the sphere formation assay, cells (1 × 10^3^) were plated onto 100-mm dishes for 5-10 days. Sphere was counted with a diameter > 20 μm.

### Tube formation assay

For the tube formation assay, 96-wells plates were coated with 100 μL of Matrigel (BD Biosciences, San Jose, CA, USA) prepared according to manufacturer's instructions [56]. Total tube numbers were counted and compared from three different fields using inverted microscope. Three independent experiment, were analyzed for statistical significance using the student's t-test. Differences were considered to be statistically significant at *p<0.05.

### Three-dimensional cultures

24-well plate pre-coated with 60 μL of Matrigel (BD Biosciences, San Jose, CA, USA) was prepared according to manufacturer's instructions [57]. Then, 50-100 cells of A498 and MDA-MB-231 cells suspended in 600 μL of growth medium with Matrigel were seeded on the top of the Matrigel layers. After incubation in 37°C to form colonies, colonies were taken a photo per 5 days.

### Immunocytochemistry

Cells were fixed with 4% paraformaldehyde and permeabilized with 0.25% triton X-100 in PBS. To detect specific molecule, cells were incubated with the appropriate primary antibodies in a solution of PBS with 1% bovine serum albumin at 4°C overnight. Antibodies used were as follows: human anti-E-cadherin (mouse monoclonal antibody, 1:200) and anti-Vimentin (rabbit polyclonal antibody, 1:200). Secondary antibodies were used with anti-mouse or anti-rabbit Alexa Fluor 488 and 555-conjugated (1:500) for 1 hour in the dark. Nuclei were counterstained using 4, 6-diamidino-2-phenylindole (DAPI) mounting medium (Vector Laboratories, Burlingame, CA, USA). Stained cells were visualized with a fluorescence microscope (Zeiss LSM-710, Munchen, Germany).

### Immunohistochemistry

The human cancer tissue was washed with TBS-T buffer and blocked with blocking solution (PBS, 5% BSA). Primary antibody staining was performed using anti-IL-4 (1:200) overnight at 4°C. After washing, ABC Kit and DAB (Vector laboratory, Burlingame, CA, USA) prepared according to manufacturer's instructions. Immuno-staining was observed and photographed using a cellSens (Olympus, Shinjuku-ku, Tokyo, Japan).

### Tumor cell implantation

For *in vivo* tumor experiments, sh-control or sh-IL-4-transfected metastatic MDA-MB-231 cells (1×10^6^ cells/mouse) were injected into fourth mammary fat pad of BALB/c nude mice (8-week-old female) (n = 5/group; Orient, Gyeonggido, Korea) to form primary tumors. Additionally, the treatment of fractionated IR (2.5Gy x 3 times) was performed at 15, 16, and 17 days after injection of cells. After 21 days injection of cells, the mice were anesthetized intraperitoneally and sacrificed. Tumor and metastatic lungs were excised from mice. Lung metastasis was analyzed by counting the number of foci in the lung. The tumor and lungs were fixed with formalin and embedded for immunohistochemistry (IHC) or were used for western blot analysis. This study was reviewed and approved by the Institutional Animal Care and Use Committee (IACUC) of Korea Institute of Radiological & Medical Science.

### Statistical analysis

All results were analyzed for statistical significance that was assessed using a Student's t-test. A P value of p < 0.05 compare with the control was considered statistically significant.

## SUPPLEMENTARY FIGURES



## References

[1] Ho JN, Kang GY, Lee SS, Kim J, Bae IH, Hwang SG, Um HD (2010). Bcl-XL and STAT3 mediate malignant actions of gamma-irradiation in lung cancer cells. Cancer science.

[2] Cheng JC, Chou CH, Kuo ML, Hsieh CY (2006). Radiation-enhanced hepatocellular carcinoma cell invasion with MMP-9 expression through PI3K/Akt/NF-kappaB signal transduction pathway. Oncogene.

[3] Martinou M, Giannopoulou E, Malatara G, Argyriou AA, Kalofonos HP, Kardamakis D (2011). Ionizing radiation affects epidermal growth factor receptor signalling and metalloproteinase secretion in glioma cells. Cancer genomics & proteomics.

[4] Camphausen K, Moses MA, Beecken WD, Khan MK, Folkman J, O'Reilly MS (2001). Radiation therapy to a primary tumor accelerates metastatic growth in mice. Cancer research.

[5] Bonner JA, Trummell HQ, Willey CD, Plants BA, Raisch KP (2009). Inhibition of STAT-3 results in radiosensitization of human squamous cell carcinoma. Radiotherapy and oncology.

[6] Maddirela DR, Kesanakurti D, Gujrati M, Rao JS (2013). MMP-2 suppression abrogates irradiation-induced microtubule formation in endothelial cells by inhibiting alphavbeta3-mediated SDF-1/CXCR4 signaling. International journal of oncology.

[7] Moeller BJ, Cao Y, Li CY, Dewhirst MW (2004). Radiation activates HIF-1 to regulate vascular radiosensitivity in tumors: role of reoxygenation, free radicals, and stress granules. Cancer cell.

[8] Timke C, Zieher H, Roth A, Hauser K, Lipson KE, Weber KJ, Debus J, Abdollahi A, Huber PE (2008). Combination of vascular endothelial growth factor receptor/platelet-derived growth factor receptor inhibition markedly improves radiation tumor therapy. Clinical cancer research.

[9] Clemenson C, Chargari C, Deutsch E (2013). Combination of vascular disrupting agents and ionizing radiation. Critical reviews in oncology/hematology.

[10] Garcia-Barros M, Paris F, Cordon-Cardo C, Lyden D, Rafii S, Haimovitz-Friedman A, Fuks Z, Kolesnick R (2003). Tumor response to radiotherapy regulated by endothelial cell apoptosis. Science.

[11] Ong ZY, Gibson RJ, Bowen JM, Stringer AM, Darby JM, Logan RM, Yeoh AS, Keefe DM (2010). Pro-inflammatory cytokines play a key role in the development of radiotherapy-induced gastrointestinal mucositis. Radiation oncology.

[12] Di Maggio FM, Minafra L, Forte GI, Cammarata FP, Lio D, Messa C, Gilardi MC, Bravata V (2015). Portrait of inflammatory response to ionizing radiation treatment. Journal of inflammation.

[13] Ruegg C, Monnier Y, Kuonen F, Imaizumi N (2011). Radiation-induced modifications of the tumor microenvironment promote metastasis. Bulletin du cancer.

[14] Bankaitis KV, Fingleton B (2015). Targeting IL4/IL4R for the treatment of epithelial cancer metastasis. Clin Exp Metastasis.

[15] Sokol CL, Barton GM, Farr AG, Medzhitov R (2008). A mechanism for the initiation of allergen-induced T helper type 2 responses. Nature immunology.

[16] Venmar KT, Carter KJ, Hwang DG, Dozier EA, Fingleton B (2014). IL4 receptor ILR4alpha regulates metastatic colonization by mammary tumors through multiple signaling pathways. Cancer research.

[17] Koller FL, Hwang DG, Dozier EA, Fingleton B (2010). Epithelial interleukin-4 receptor expression promotes colon tumor growth. Carcinogenesis.

[18] Todaro M, Lombardo Y, Francipane MG, Alea MP, Cammareri P, Iovino F, Di Stefano AB, Di Bernardo C, Agrusa A, Condorelli G, Walczak H, Stassi G (2008). Apoptosis resistance in epithelial tumors is mediated by tumor-cell-derived interleukin-4. Cell death and differentiation.

[19] Ruffell B, Affara NI, Coussens LM (2012). Differential macrophage programming in the tumor microenvironment. Trends in immunology.

[20] He L, Hannon GJ (2004). MicroRNAs: small RNAs with a big role in gene regulation. Nature reviews Genetics.

[21] Hwang HW, Mendell JT (2006). MicroRNAs in cell proliferation, cell death, and tumorigenesis. British journal of cancer.

[22] Zhang W, Liu J, Wang G (2014). The role of microRNAs in human breast cancer progression. Tumour biology.

[23] Miyatake S, Nonoguchi N, Furuse M, Yoritsune E, Miyata T, Kawabata S, Kuroiwa T (2015). Pathophysiology, diagnosis, and treatment of radiation necrosis in the brain. Neurol Med Chir (Tokyo).

[24] Dai T, Shah MA (2015). Chemoradiation in oesophageal cancer. Best Pract Res Clin Gastroenterol.

[25] Zhang S, Zheng X, Huang H, Wu K, Wang B, Chen X, Ma S (2015). Afatinib increases sensitivity to radiation in non-small cell lung cancer cells with acquired EGFR T790M mutation. Oncotarget.

[26] McBride WH, Chiang CS, Olson JL, Wang CC, Hong JH, Pajonk F, Dougherty GJ, Iwamoto KS, Pervan M, Liao YP (2004). A sense of danger from radiation. Radiat Res.

[27] Shin JW, Son JY, Raghavendran HR, Chung WK, Kim HG, Park HJ, Jang SS, Son CG (2011). High-dose ionizing radiation-induced hematotoxicity and metastasis in mice model. Clin Exp Metastasis.

[28] Chang Y, Xu L, An H, Fu Q, Chen L, Lin Z, Xu J (2015). Expression of IL-4 and IL-13 predicts recurrence and survival in localized clear-cell renal cell carcinoma. International journal of clinical and experimental pathology.

[29] Gyorffy B, Schafer R (2009). Meta-analysis of gene expression profiles related to relapse-free survival in 1,079 breast cancer patients. Breast cancer research and treatment.

[30] Angiogenesis. Folkman J (2006). Annu Rev Med.

[31] Piva M, Domenici G, Iriondo O, Rabano M, Simoes BM, Comaills V, Barredo I, Lopez-Ruiz JA, Zabalza I, Kypta R, Vivanco M (2014). Sox2 promotes tamoxifen resistance in breast cancer cells. EMBO molecular medicine.

[32] Wang D, Lu P, Zhang H, Luo M, Zhang X, Wei X, Gao J, Zhao Z, Liu C (2014). Oct-4 and Nanog promote the epithelial-mesenchymal transition of breast cancer stem cells and are associated with poor prognosis in breast cancer patients. Oncotarget.

[33] Kim K, Ro JY, Kim S, Cho YM (2012). Expression of stem-cell markers OCT-4 and CD133: important prognostic factors in papillary renal cell carcinoma. Human pathology.

[34] Leis O, Eguiara A, Lopez-Arribillaga E, Alberdi MJ, Hernandez-Garcia S, Elorriaga K, Pandiella A, Rezola R, Martin AG (2012). Sox2 expression in breast tumours and activation in breast cancer stem cells. Oncogene.

[35] Kim RK, Suh Y, Yoo KC, Cui YH, Hwang E, Kim HJ, Kang JS, Kim MJ, Lee YY, Lee SJ (2015). Phloroglucinol suppresses metastatic ability of breast cancer cells by inhibition of epithelial-mesenchymal cell transition. Cancer science.

[36] Chaudhry MA, Omaruddin RA, Brumbaugh CD, Tariq MA, Pourmand N (2013). Identification of radiation-induced microRNA transcriptome by next-generation massively parallel sequencing. J Radiat Res.

[37] Weidhaas JB, Babar I, Nallur SM, Trang P, Roush S, Boehm M, Gillespie E, Slack FJ (2007). MicroRNAs as potential agents to alter resistance to cytotoxic anticancer therapy. Cancer research.

[38] Antonov AV, Knight RA, Melino G, Barlev NA, Tsvetkov PO (2013). MIRUMIR: an online tool to test microRNAs as biomarkers to predict survival in cancer using multiple clinical data sets. Cell death and differentiation.

[39] Barcellos-Hoff MH, Park C, Wright EG (2005). Radiation and the microenvironment- tumorigenesis and therapy. Nature reviews Cancer.

[40] Kawakami M, Kawakami K, Stepensky VA, Maki RA, Robin H, Muller W, Husain SR, Puri RK (2002). Interleukin 4 receptor on human lung cancer: a molecular target for cytotoxin therapy. Clinical cancer research.

[41] Todaro M, Zerilli M, Ricci-Vitiani L, Bini M, M Perez Alea, A Maria Florena, Miceli L, Condorelli G, Bonventre S, Di Gesu G, De Maria R, Stassi G (2006). Autocrine production of interleukin-4 and interleukin-10 is required for survival and growth of thyroid cancer cells. Cancer research.

[42] Roca H, Craig MJ, Ying C, Varsos ZS, Czarnieski P, Alva AS, Hernandez J, Fuller D, Daignault S, Healy PN, Pienta KJ (2012). IL-4 induces proliferation in prostate cancer PC3 cells under nutrient-depletion stress through the activation of the JNK-pathway and survivin up-regulation. J Cell Biochem.

[43] Gocheva V, Wang HW, Gadea BB, Shree T, Hunter KE, Garfall AL, Berman T, Joyce JA (2010). IL-4 induces cathepsin protease activity in tumor-associated macrophages to promote cancer growth and invasion. Genes Dev.

[44] Surana R, Wang S, Xu W, Jablonski SA, Weiner LM (2014). IL4 limits the efficacy of tumor-targeted antibody therapy in a murine model. Cancer Immunol Res.

[45] Francipane MG, Alea MP, Lombardo Y, Todaro M, Medema JP, Stassi G (2008). Crucial role of interleukin-4 in the survival of colon cancer stem cells. Cancer research.

[46] Zhan M, Han ZC (2004). Phosphatidylinositide 3-kinase/AKT in radiation responses. Histol Histopathol.

[47] Li BH, Yang XZ, Li PD, Yuan Q, Liu XH, Yuan J, Zhang WJ (2008). IL-4/Stat6 activities correlate with apoptosis and metastasis in colon cancer cells. Biochemical and biophysical research communications.

[48] Venmar KT, Fingleton B (2014). Lessons from immunology: IL4R directly promotes mammary tumor metastasis. Oncoimmunology.

[49] Vandenboom Ii TG, Li Y, Philip PA, Sarkar FH (2008). MicroRNA and Cancer: Tiny Molecules with Major Implications. Current genomics.

[50] Esquela-Kerscher A, Slack FJ (2006). Oncomirs- microRNAs with a role in cancer. Nature reviews Cancer.

[51] Mott JL, Kobayashi S, Bronk SF, Gores GJ (2007). mir-29 regulates Mcl-1 protein expression and apoptosis. Oncogene.

[52] Li J, Du L, Yang Y, Wang C, Liu H, Wang L, Zhang X, Li W, Zheng G, Dong Z (2013). MiR-429 is an independent prognostic factor in colorectal cancer and exerts its anti-apoptotic function by targeting SOX2. Cancer letters.

[53] Park SY, Lee JH, Ha M, Nam JW, Kim VN (2009). miR-29 miRNAs activate p53 by targeting p85 alpha and CDC42. Nature structural & molecular biology.

[54] Chung HJ, Choi YE, Kim ES, Han YH, Park MJ, Bae IH (2015). miR-29b attenuates tumorigenicity and stemness maintenance in human glioblastoma multiforme by directly targeting BCL2L2. Oncotarget.

[55] Lee WS, Woo EY, Kwon J, Park MJ, Lee JS, Han YH, Bae IH (2013). Bcl-w Enhances Mesenchymal Changes and Invasiveness of Glioblastoma Cells by Inducing Nuclear Accumulation of beta-Catenin. PloS one.

[56] Zheng X, Chopp M, Lu Y, Buller B, Jiang F (2013). MiR-15b and miR-152 reduce glioma cell invasion and angiogenesis via NRP-2 and MMP-3. Cancer letters.

[57] Angelucci A, Gravina GL, Rucci N, Millimaggi D, Festuccia C, Muzi P, Teti A, Vicentini C, Bologna M (2006). Suppression of EGF-R signaling reduces the incidence of prostate cancer metastasis in nude mice. Endocrine-related cancer.

