# Supplementary material for: IL-4, a direct target of miR-340/429, is involved in radiation-induced aggressive tumor behavior in human carcinoma cells
Source: Oncotarget. 2016 Nov 24;7(52):86836–56. doi: 10.18632/oncotarget.13561 (PMC5349958; doi:10.18632/oncotarget.13561)
Supplement: Supplementary file 1 [file oncotarget-07-86836-s001.pdf]

IL-4, a direct target of miR-340/429, is involved in radiation-induced aggressive tumor behavior in human carcinoma cells

SUPPLEMENTARY FIGURES

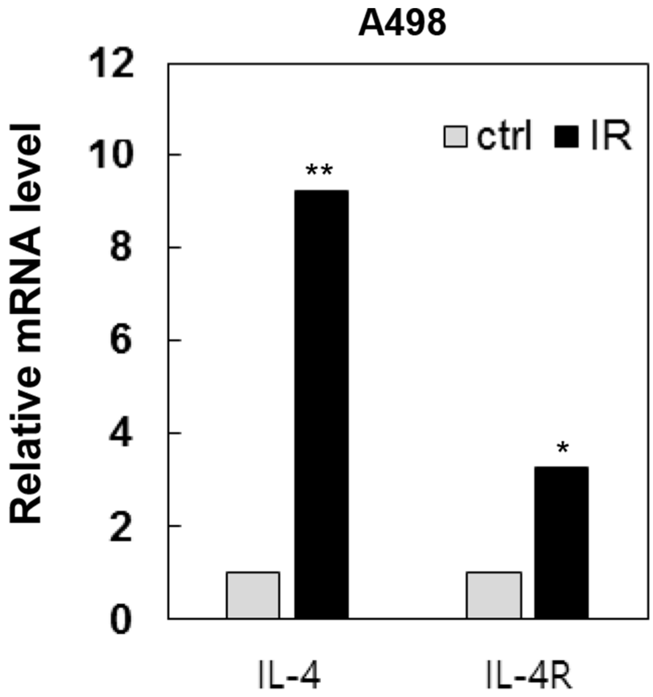

Supplementary Figure S1: IR increases IL-4 and IL-4Rα expressions in A498 cells. The level of IL-4 and IL-4R mRNA were measured in A498 cells by qRT-PCR after exposure to IR (5 Gy) for 1 hour. The data are presented as the mean S.D. (* $P < 0.05$, ** $P < 0.005$, Student's t-test).

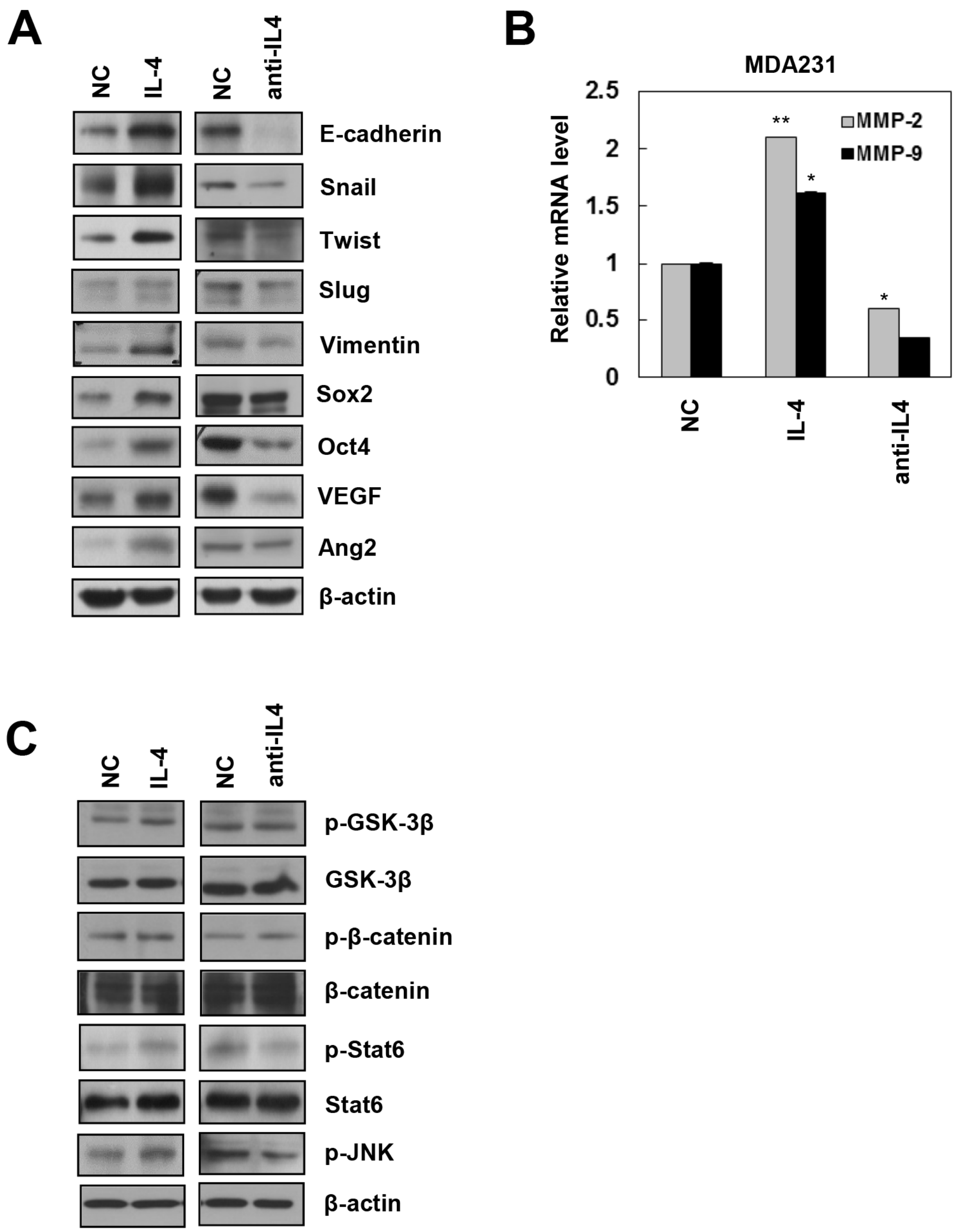

Supplementary Figure S2: Recombinant IL-4 or neutralizing of IL-4 antibody regulates EMT, migration, invasion, angiogenesis, and stemness in MDA-MB-231 cells. Cells were treated with recombinant IL-4 (0.5 ng/mL) or anti-IL-4 antibody (10 µg/mL) for 24 hours. **A.** EMT, angiogenesis, and stemness associated protein expression levels were detected by Western blot analysis. **B.** The MMP-2/9 mRNA levels were determined by qRT-PCR. The data are presented as the mean S.D. (* $P < 0.05$, ** $P < 0.005$, Student's t-test). **C.** The expression of IL-4-induced signaling molecules was detected by Western blot analysis.

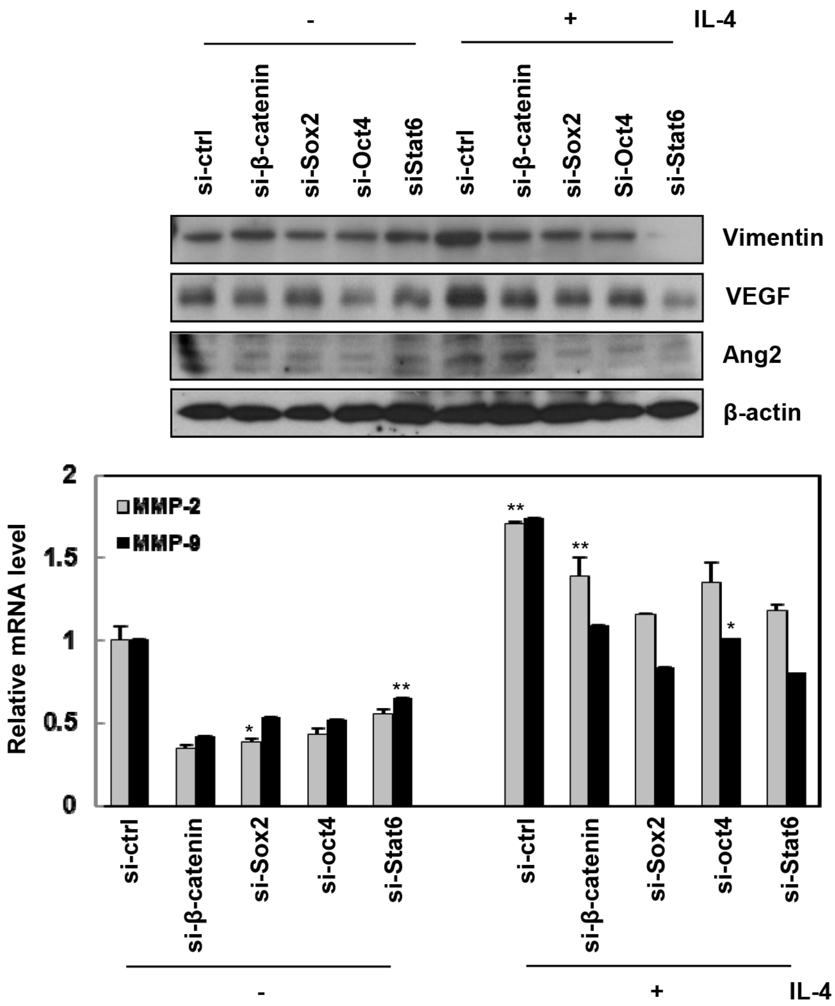

Supplementary Figure S3: IL-4 activates JNK-β-catenin/Stat6 signaling pathway. A498 cells were transfected with siRNA against indicated signaling molecules, and vimentin, VEGF, Ang2, and MMP-2/9 levels were determined by Western blot analysis (top) and qRT-PCR (bottom), respectively. The data are presented as the mean S.D. (* $P < 0.05$, ** $P < 0.005$, Student's t-test).

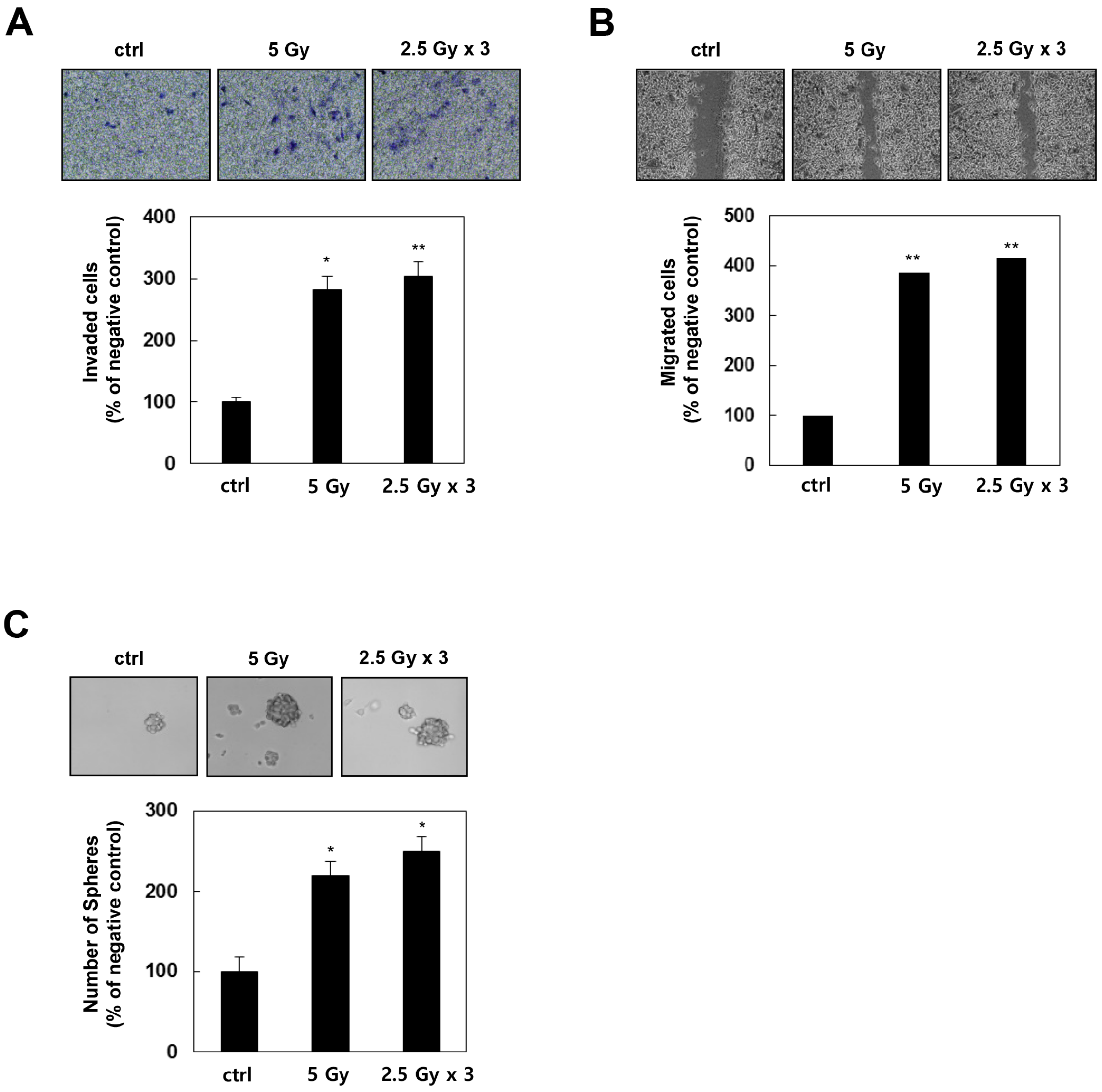

Supplementary Figure S4: Phenomena of a single dose (5Gy)-exposed cells are similar to those of fractionated dose (2.5Gy x 3 times)-treated cells. After MDA-MB-231 cells were treated with a single dose (5Gy) or fractionated dose (2.5Gy x 3 times) IR, these cells were examined wound healing, invasion, and sphere formation assay. The data are presented as the mean S.D. (* $P < 0.05$, ** $P < 0.005$, Student's t-test).

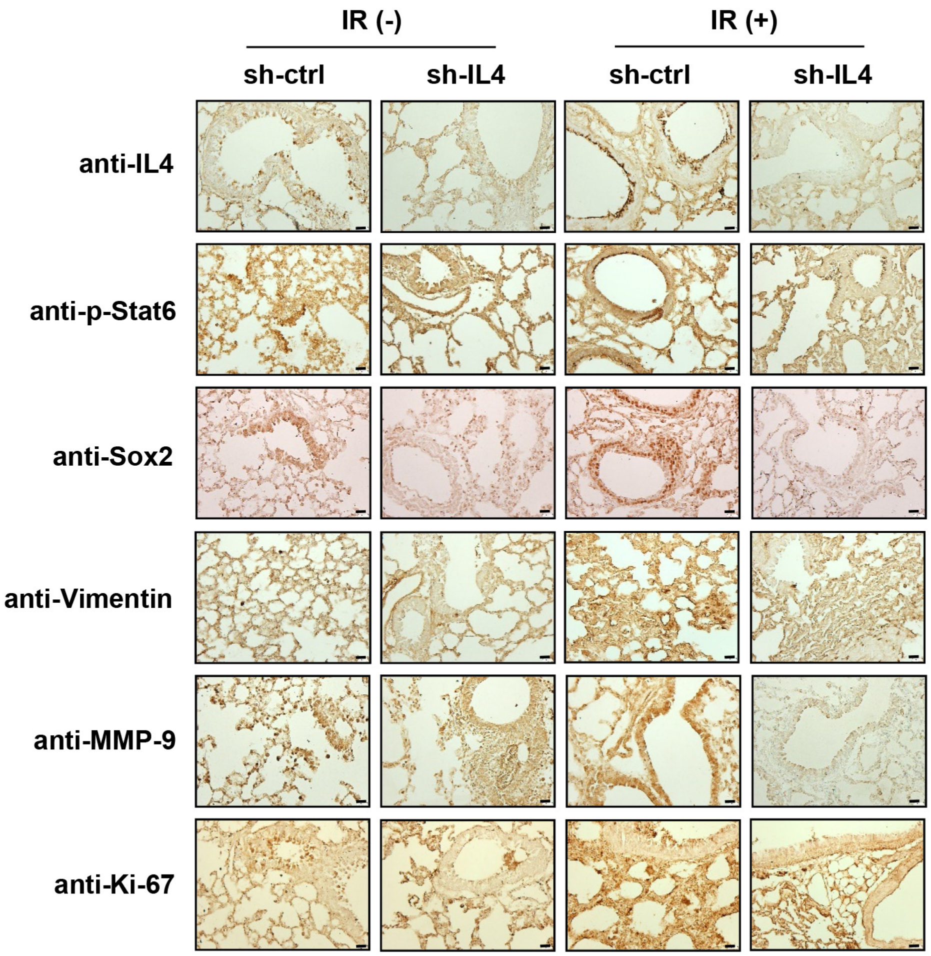

Supplementary Figure S5: IR-induced IL-4-related signaling components are promoted in lung metastatic tissues. Expressions of p-Stat6, Sox2, Vimentin, MMP-9, and Ki-67 were verified in mouse lung tissues by IHC analysis (Scale bar, 20 μm).
